# Taxonomic status of *Apostolepisbarrioi* Lema, 1978, with comments on the taxonomic instability of *Apostolepis* Cope, 1862 (Serpentes, Dipsadidae)

**DOI:** 10.3897/zookeys.841.33404

**Published:** 2019-04-23

**Authors:** Omar Machado Entiauspe-Neto, Arthur de Sena, Arthur Tiutenko, Daniel Loebmann

**Affiliations:** 1 Universidade Federal do Rio Grande, Instituto de Ciências Biológicas, Laboratório de Vertebrados, Av. Itália Km 8, CEP: 96203-900, Vila Carreiros, Rio Grande, Rio Grande do Sul, Brazil Universidade Federal do Rio Grande Rio Grande Brazil; 2 Universidade do Estado de Mato Grosso, Departamento de Biologia. CEP: 78690-000, Nova Xavantina, Mato Grosso, Brazil Universidade do Estado de Mato Grosso Nova Xavantina Brazil; 3 University of Erlangen-Nuremberg, Schlossplatz 6, D-91054 Erlangen, Germany University of Erlangen-Nuremberg Erlangen Germany

**Keywords:** Elapomorphini, Neotropical, synonymy, taxonomy

## Abstract

*Apostolepis* is a diverse neotropical snake genus, which has been historically subjected to poor taxonomic descriptions, largely based on either a small type series or subjective diagnoses. We evaluate the case of *Apostolepisbarrioi* Lema, 1978 and its intricate taxonomic history, suggesting its synonymization with *Apostolepisdimidiata* (Jan, 1862), and providing brief commentary on the taxonomic instability that has been plaguing the genus.

## Introduction

The Neotropical dipsadid snake genus *Apostolepis* Cope, 1862 comprises over 30 species, with an even broader synonym list, being marked by a systemic proliferation of “poorly defined” taxa, described based on single or few individuals with poor documentation of variation (Vanzolini 1986; Ferrarezzi 1993; Harvey 1999; Ferrarezzi et al. 2005; Nogueira et al. 2012). *Apostolepisdimidiata* (Jan, 1862) is a small-sized fossorial snake that occurs in the Cerrado, Chaco and Atlantic Forests at Argentina, Brazil and Paraguay (Cei 1993; Giraudo and Scrocchi 1998; Harvey 1999). Jan (1862) described *Elapomorphusdimidiatus* based on a specimen from “Brazil”, and allocated it to the subgenus Elapomojus Jan, 1862. Later, Peters (1880) described *Elapomorphuserythronotus* based on a specimen from “São Paulo” in southeastern Brazil. Cope (1887) presented two brief taxon descriptions, *Apostolepiserythronotuslineatus* and *Rhynchonyxambinigervittatus*, both from Chapada dos Guimarães, Mato Grosso, in central-western Brazil. Boulenger (1896) elevated both of Cope’s subspecies to species level and placed *Elapomorphuserythronotus* in *Apostolepis*. Werner (1897) described *Apostolepisnigriceps* based on two specimens, of which only one has a known locality given as “São Paulo”, in southeastern Brazil. Lema (1978) described three new species for Paraguay: *Apostolepisbarrioi* from the Ypané River, Cororo, Concepcíon Province; *Apostolepisventrimaculatus* from “Paraguay”; and *Apostolepisvillaricae* from Villa Rica, Concepcíon Province. Later, Lema (1986) would synonymize *A.erythronota*, *A.nigriceps* and *A.ventrimaculatus* with *A.dimidiata*, while also revalidating *A.lineata*. Lema (1993) presented a review on the morphological variation of *A.dimidiata*, while also allocating the species he previously described, *A.barrioi* and *A.ventrimaculatus*, as synonyms of the former.

## Taxonomy

Recently, Cabral et al. (2017) presented a revalidation of *A.barrioi*, diagnosing it from all congeners based on an immaculate white venter, narrow dorsolateral stripes not in contact with the ventrals, and a terminal black shield. There is also a wide overlap between the meristic variation of *A.barrioi* and *A.dimidiata*, such as in the number of ventral scales (222–256 in *A.barrioi*; 214–264 in *A.dimidiata*) and subcaudal scales (23–55 in *A.barrioi*; 22–39 in *A.dimidiata*), in its morphometric variation (given in mm), in snout-vent length (188–542 in *A.barrioi*; 180–676 in *A.dimidiata*) and tail length (16–45 in *A.barrioi*; 16–60 in *A.dimidiata*), and geographic variation, since both species are sympatric along their whole distribution, as reported by the authors (Cabral et al. 2017: 246). Furthermore, the authors present a comparative table of *Apostolepis* species in which *A.barrioi* is stated as having an immaculate venter and *A.dimidiata*, a venter heavily pigmented with black, having only the edge of the ventrals white. This is in clear conflict with the original description, considering that the holotype of *A.dimidiata* presented an immaculate yellow venter according to the original description “[...] *parte inferiore del corpo é giallastra, meno la testa che inferiormente ha del nero sugli inframascellari e sulle squame che stanno in vicinanzi ai sottolabiali*” (En: lower part of the body is yellowish, except for the head, that has black inferiorly, in the inframaxillary (region) and in the scales near the infralabials) (Jan 1862: 48). Unfortunately, this specimen (holotype of *A.dimidiata*) could not be examined, since it was destroyed during the Second World War. It is also relevant that Lema (1993: 47) presents a plate that encompasses all known ventral pattern variation for *A.dimidiata*, with a gradual change from immaculate yellow to black ventral patterns.

**Figure 1.**
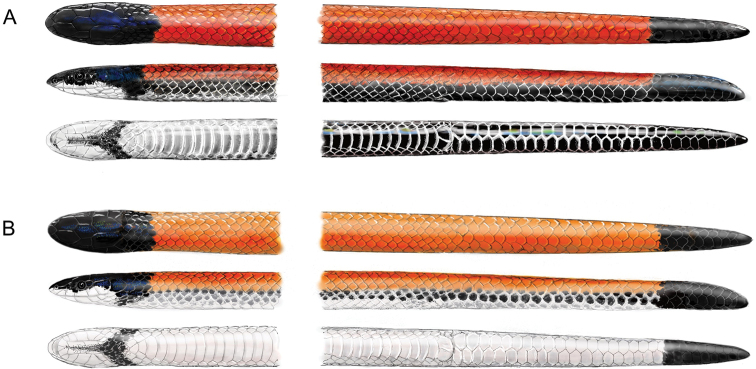
Dorsal, lateral and ventral illustrations of previously recognized taxa, *Apostolepisdimidiata* (**A**) and *A.barrioi* (**B**), according to the diagnoses of Cabral et al. (2017). However, these represent merely phenotypic variations of *A.dimidiata* and, according to the descriptions of Jan (1862) and Lema (1978), both holotypes of *A.barrioi* and *A.dimidiata* present the bottom coloration.

Considering that *Apostolepisbarrioi* and *A.dimidiata* share the same morphological features and variation (Fig. 1), present virtually identical geographic distribution, and both descriptions are based on specimens that have the same ventral coloration, which was erroneously cited as “diagnostic” at the time, we argue that *A.barrioi* Lema, 1978 should be relegated as a junior synonym of *A.dimidiata* (Jan, 1862). The work of Cabral et al. (2017) seems to follow a recent, genus-wide trend, in which several species have been described based on poorly supported diagnoses (e.g. *Apostolepismariae*Borges-Nojosa et al. 2017 (2016); *A.roncadori* Lema, 2016; *A.thalesdelemai* Borges-Nojosa et al. 2016 (2017); *A.underwoodi* Lema & Campbell, 2017). None of these studies presented descriptions of osteology or hemipenes, nor do they include molecular support for their proposed species, relying exclusively upon highly variable morphological characters such as coloration and body shape. It is also noteworthy that, *A.barrioi* was described, synonymized, and then revalidated by the same author over a timespan of almost 40 years.

## Final remarks

**Figure 2.**
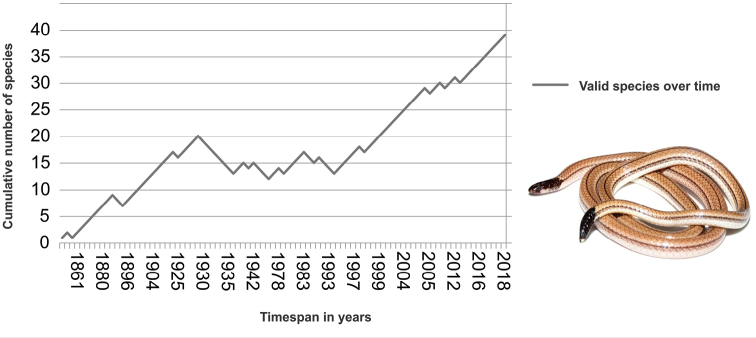
The impact of the taxonomic history of *Apostolepis* Cope, 1862 species. The line refers to the cumulative number of species considered as valid during the time span, suffering either reductions from synonymies or additions from descriptions and revalidations. Inset picture: *Apostolepis* sp. from Serra do Cachimbo, Pará, Brazil.

Unfortunately, several interest conflicts among researchers in the past decades have caused strong instability in *Apostolepis*, as well as most Elapomorphini taxa (Fig. 2). Here we present an overview of the currently recognized species of *Apostolepis* as well as their known specimens and diagnoses. It is noteworthy that poor diagnoses and small type series are usually associated with previous synonymizations (Table 1). We urge our fellow authors not to commit taxonomic malpractice and to carefully generate, rethink and analyze their data, providing compelling evidence for their claims. The careless proliferation and splitting of taxa may present deleterious consequences not only to the field of taxonomy but also to directing conservation efforts. An integrative revision, preferably incorporating aspects of external and internal morphology, along with molecular data, is largely warranted in order to mitigate and reevaluate the taxonomy of *Apostolepis* as a whole.

**Table 1.** Valid species of *Apostolepis* Cope, 1862 up to date. Characters present on diagnosis: Coloration (CO), Meristic (ME), Morphometric (MO), Subjective character states related to external morphology (SU), Molecular Data (MD), Internal Morphology (IM). References: ^1^França et al. 2018; ^2^Lema 2002b; ^3^Peters 1869; ^4^Ferrarezzi et al. 2005; ^5^Rodrigues 1993; ^6^Reinhardt 1861; ^7^Peracca 1904; ^8^Harvey et al. 2001; ^9^Gomes 1915; ^10^Lema 2003; ^11^Lema 2002; ^12^Jan 1862; ^13^Schlegel 1837; ^14^Duméril et al. 1854; ^15^Prado 1942; ^16^Koslowsky 1898; ^17^Santos et al. 2018; ^18^Cope 1887; ^19^Gomes in Amaral 1921; ^20^Borges-Nojosa et al. 2017; ^21^Harvey 1999; ^22^Lema and Renner 2004; ^23^Amaral 1935; ^24^Peters 1869; ^25^Boulenger 1896; ^26^Amaral 1922; ^27^Boulenger 1903; ^28^Giraudo and Scrocchi 1998; ^29^Lema and Renner 2006; ^30^Lema 2004a; ^31^Ruthven 1927; ^32^Lema 2004b; ^33^Lema 2016; ^34^Lema and Campbell 2017.

Taxon	Year of description	Individuals in type series	Previously synonymized?	CO	ME	MO	SU	MD	IM
* Apostolepis adhara * ^1^	2018	2	No	+	+	+	-	-	+
* Apostolepis albicollaris * ^2^	2002	28	No	+	+	+	+	-	-
* Apostolepis ambinigra * ^3^	1869	1	No	+	+	+	-	-	-
* Apostolepis ammodites * ^4^	2005	25	No	+	+	+	+	-	+
* Apostolepis arenaria * ^5^	1993	4	No	+	+	+	-	-	-
* Apostolepis assimilis * ^6^	1861	1 (≥)	No	+	+	-	-	-	-
* Apostolepis borelli * ^7^	1904	1	Yes	+	+	+	-	-	-
* Apostolepis breviceps * ^8^	2001	4	No	+	+	+	+	-	-
* Apostolepis cearensis * ^9^	1915	7	No	+	+	+	-	-	-
* Apostolepis cerradoensis * ^10^	2003	1	No	+	+	+	+	-	-
* Apostolepis christineae * ^11^	2002	1	No	+	+	+	+	-	-
* Apostolepis dimidiata * ^12^	1862	1	Yes	+	+	-	+	-	-
* Apostolepis dorbignyi * ^13^	1837	1	No	+	+	+	-	-	-
* Apostolepis flavotorquata * ^14^	1854	1	Yes	+	+	+	-	-	-
* Apostolepis gaboi * ^5^	1993	1	No	+	+	+	-	-	-
* Apostolepis goiasensis * ^15^	1942	1	Yes	+	+	+	+	-	-
* Apostolepis intermedia * ^16^	1898	1	No	+	+	+	-	-	-
* Apostolepis kikoi * ^17^	2018	5	No	+	+	+	-	-	+
* Apostolepis lineata * ^18^	1887	1	Yes	+	-	-	-	-	-
* Apostolepis longicaudata * ^19^	1921	1	No	+	+	+	+	-	-
* Apostolepis mariae * ^20^	2017	22	No	+	+	+	+	-	-
* Apostolepis multicincta * ^21^	1999	3	No	+	+	+	-	-	-
* Apostolepis nelsonjorgei * ^22^	2004	7	No	+	+	+	+	-	-
* Apostolepis niceforoi * ^23^	1935	1	No	+	+	+	+	-	-
* Apostolepis nigrolineata * ^24^	1869	1	Yes	+	+	+	-	-	-
* Apostolepis nigroterminata * ^25^	1896	1	No	+	+	+	-	-	-
* Apostolepis phillipsi * ^21^	1999	1	No	+	+	+	-	-	-
* Apostolepis polylepis * ^26^	1922	4	No	+	+	+	+	-	-
* Apostolepis pymi * ^27^	1903	1	Yes	+	+	+	-	-	-
* Apostolepis quinquelineata * ^25^	1896	1	Yes	+	+	+	-	-	-
* Apostolepis quirogai * ^28^	1998	2	No	+	+	+	-	-	-
* Apostolepis serrana * ^29^	2006	1	No	+	+	+	+	-	-
* Apostolepis striata * ^30^	2004a	1	No	+	+	+	+	-	-
* Apostolepis tenuis * ^31^	1927	1	Yes	+	+	+	-	-	-
* Apostolepis tertulianobeui * ^32^	2004b	1	No	+	+	+	+	-	-
* Apostolepis thalesdelemai * ^20^	2017	15	No	+	+	+	+	-	-
* Apostolepis roncadori * ^33^	2016	1	No	+	+	+	+	-	-
* Apostolepis underwoodi * ^34^	2017	3	No	+	+	+	+	-	-
* Apostolepis vittata * ^18^	1887	1	Yes	+	-	-	-	-	-
